# Childhood sexual abuse in patients with severe mental Illness: Demographic, clinical and functional correlates

**DOI:** 10.1111/acps.13302

**Published:** 2021-04-10

**Authors:** Nomi Werbeloff, Johan Hilge Thygesen, Joseph F. Hayes, Essi M. Viding, Sonia Johnson, David P.J. Osborn

**Affiliations:** ^1^ The Louis and Gabi Weisfeld School of Social Work Bar Ilan University Ramat Gan Israel; ^2^ Division of Psychiatry University College London London UK; ^3^ Camden and Islington NHS Foundation Trust London UK; ^4^ Institute of Health Informatics University College London London UK; ^5^ Division of Psychology & Language Sciences University College London London UK

**Keywords:** childhood trauma, psychotic disorders, sexual abuse

## Abstract

**Objective:**

To use data from electronic health records (EHRs) to describe the demographic, clinical and functional correlates of childhood sexual abuse (CSA) in patients with severe mental illness (SMI), and compare their clinical outcomes (admissions and receipt of antipsychotic medications) to those of patients with no recorded history of CSA.

**Methods:**

We applied a string‐matching technique to clinical text records of 7000 patients with SMI (non‐organic psychotic disorders or bipolar disorder), identifying 619 (8.8%) patients with a recorded history of CSA. Data were extracted from both free‐text and structured fields of patients’ EHRs.

**Results:**

Comorbid diagnoses of major depressive disorder, post‐traumatic stress disorder and personality disorders were more prevalent in patients with CSA. Positive psychotic symptoms, depressed mood, self‐harm, substance use and aggression were also more prevalent in this group, as were problems with relationships and living conditions. The odds of inpatient admissions were higher in patients with CSA than in those without (adjusted OR = 1.95, 95% CI: 1.64–2.33), and they were more likely to have spent more than 10 days per year as inpatients (adjusted OR = 1.32, 95% CI: 1.07–1.62). Patients with CSA were more likely to be prescribed antipsychotic medications (adjusted OR = 2.48, 95% CI: 1.69–3.66) and be given over 75% of the maximum recommended daily dose (adjusted OR = 1.72, 95% CI: 1.44–2.04).

**Conclusion:**

Data‐driven approaches are a reliable, promising avenue for research on childhood trauma. Clinicians should be trained and skilled at identifying childhood adversity in patients with SMI, and addressing it as part of the care plan.

**Significant outcomes**

- The rates of CSA recorded in electronic health records of people with SMI are considerably lower than self‐reported rates found in previous studies.
- Exposure to CSA is associated with more severe psychiatric symptomatology, impaired functioning and higher rates of comorbid major depressive disorder, post‐traumatic stress disorder and personality disorders.
- Higher odds and longer duration of inpatient admissions, as well as higher dosage of antipsychotic medication, are evident in people with SMI and a history of CSA as compared to those without such a documented history.

**Limitations**

- A string‐matching technique was used to identify cases of CSA in clinical text notes. While the positive predictive value was high, this does not account for the possibility that some cases may have been missed.
- Patients with more or longer admissions might have had more opportunities to have CSA recorded in their EHRs, partially explaining some of the associations found in this study.
- Our search strategy did not allow us to extract information on the age or duration of sexual abuse, variables which have been shown to be associated with the clinical presentation of psychotic disorders.

## 1 INTRODUCTION

There are many definitions of childhood sexual abuse (CSA), encompassing a range of sexually abusive acts towards children. These include sexual assault, rape, incest and the commercial sexual exploitation of children.^1^ A meta‐analyses of 331 global prevalence studies reported a total combined prevalence of 11.8%, with 7.6% of males and 18% of females reporting a history of CSA.^2^ A recent review suggests that CSA is associated with a range of long‐term outcomes, including psychosocial, psychiatric and physical health outcomes.^3^

Consistent evidence suggests that there is an association between childhood trauma (CT), particularly sexual and physical abuse, and later psychotic disorders.^4^ A meta‐analysis of 23 studies that measured CT using psychometric instruments found that the prevalence of childhood sexual abuse in patients with psychosis is estimated at 26.3% (95% CI: 21.2% to 32.2%).^5^ This figure exceeds that reported for both men and women in a meta‐analysis of community and student samples (men: 7.9% (95% CI: 6.0–10.3), women: 19.7% (95% CI: 16.7–23.0)).^6^

Exposure to CT among people with severe mental illness (SMI)—namely schizophrenia, bipolar disorder and other non‐organic psychotic disorders—is associated with a range of negative outcomes.^7^ Specifically, patients with a history of CT report more severe positive symptoms,^8^, ^9^, ^10^ as well as increased rates of substance misuse^4^, ^11^, ^12^ and self‐harm.^9^, ^11^ Previous research also suggests that CT leads to impaired social and vocational functioning in adulthood.^13^, ^14^, ^15^ From a clinical perspective, a history of CT has been associated with less medication adherence^16^ and higher rates of readmission and relapse.^7^, ^13^

The majority of previous studies used structured questionnaires, such as the Childhood Trauma Questionnaire,^17^ to assess CSA in patients with SMI. The current study offers a novel approach to identify CSA as recorded in free‐text of electronic health records (EHRs). This approach affords the opportunity to examine real‐life recording of CSA, as well as the course of illness and longer‐term outcomes among patients with SMI in a naturalistic setting.

### 1.1 Aims of the study

We aim to describe the clinical and functional characteristics of patients with SMI and a documented history of CSA, and compare their clinical outcomes (admissions and record of antipsychotic medications) to those of patients with no recorded history of childhood sexual abuse in a large mental healthcare provider in the United Kingdom. We hypothesise that patients with SMI and a history of CSA will have more severe psychiatric symptoms and functional impairment, higher rates of comorbidities and psychiatric admissions, and higher prescribed dosages of antipsychotic medication than patients without a history of CSA.

## 2 MATERIALS AND METHODS

### 2.1 Setting and participants

Data for this study were obtained from Camden & Islington NHS Foundation Trust (C&I NHS FT) using the Clinical Record Interactive Search (CRIS) tool.^18^ CRIS is an application developed to enable routinely collected EHRs, containing both structured fields (such as dates and pick‐lists) and unstructured free‐text fields (including clinical notes and correspondence) to be used in research, using an explicit de‐identification process.^19^ C&I NHS FT is a large mental health provider serving a geographic catchment area of two inner‐city London boroughs, and approximately 470,000 residents. The database contains full but anonymised information from over 130,000 mental health service users. Studies using CRIS received ethical approval from the NRES Committee East of England – Cambridge Central (19/EE/0210).

For purposes of this study, we identified patients with non‐organic psychotic disorders or bipolar disorder (ICD‐10 diagnosis of F20‐F29 or F30‐31) who were in contact with services in the years 2009–2017 and had at least one year of follow‐up data available. Additionally, only patients with at least one recorded assessment using the Health of the Nation Outcome Scales (HoNOS) were included in the study.

### 2.2 Identification of CSA in EHRs

String matching was used to identify a list of key phrases depicting CSA in free‐text fields. Initially, a group of clinical experts defined relevant search terms. We then used an iterative process, whereby a sample of records produced by the string‐matching process was reviewed and additional key phrases were extracted from the text and added to the search strategy. Additionally, to refine the search strategy, exclusion rules were added in a similar way.

Key phrases used in the string matching process included terms such as ‘childhood sexual abuse’, ‘history of sexual abuse’, ‘csa’, ‘sexual abuse at age X’, ‘sexually abused between the ages of X‐Y’, ‘sexually abused as a child’, ‘childhood Hx of sexual abuse’ and ‘childhood experiences of sexual abuse'. Exclusion rules included phrases negating a history of CSA such as ‘no CSA’, ‘nil csa’, ‘denied a childhood history of sexual abuse’, as well as those suggesting a possible history of CSA. The full list of terms used for the identification of CSA is presented in Appendix 1. We tested the positive predictive value of this search strategy by manually reviewing 100 notes identified as positive instances of CSA and found a PPV of 95%.

### 2.3 Correlates of CSA

Demographic variables, including sex, ethnicity and marital status, were extracted from structured fields within CRIS.

Social deprivation was estimated using The Index of Multiple Deprivation (IMD). This is a measure that combines national census information from 38 indicators into seven domains of deprivation (income; employment; health and disability; education, skills and training; barriers to housing and services; living environment and crime), to create an individual score of deprivation for each area.^20^ This creates one deprivation score for 32 482 ‘lower super output areas’ in England, geographical units used for the reporting of neighbourhood‐level statistics. Each area has an average population of around 1500 people (about 400 households). Patients’ addresses are recorded in routinely collected clinical data. We obtained IMD scores by linking the lower super output area code of each patient's permanent address to 2011 national data. IMD scores were classified into quartiles.

Additional clinical and functional variables were extracted from the HoNOS—a validated instrument routinely used by professionals to describe recent health and functioning in individuals with mental health problems.^21^ The scale comprises 12 items measuring different aspects of symptoms and functioning. Each item is rated on a Likert‐style scale, ranging from 0 (no problem) to 4 (severe problem). The following items were used in this study: substance misuse, self‐harm, depressed mood, positive psychotic symptoms (hallucinations/delusions), aggressive behaviour, problems with relationship and problems with living conditions. All items were dichotomised to indicate the lifetime presence or absence of the condition/symptom at a moderate‐severe level.

Finally, we extracted information from ICD‐10‐structured fields on comorbid psychiatric diagnoses, including major depressive disorder, post‐traumatic stress disorder and personality disorders.

### 2.4 Clinical outcomes

The two primary clinical outcomes were inpatient admission to a psychiatric ward and receipt of antipsychotic medication. Data for all admissions were extracted from structured fields within CRIS to derive two binary variables: yes/no admission; and among those with at least one inpatient admission – number of admission days per year above or below the cohort median (10 days).

The use of antipsychotic medication was identified through the Natural Language Processing (NLP) application for ‘medication’ developed by the South London and Maudsley NHS Foundation Trust Biomedical Research Centre.^22^ The application was developed using a gazetteer of generic and commercial names for all medications in UK use in order to ascertain instances where the patient was reported as receiving these, with supplementary rules for ascertaining recorded dose, frequency/timing and starting/stopping statements. This application was applied to free‐text fields in the EHRs, and two binary variables were created: yes/no record of receipt of antipsychotic medication; and among those receiving antipsychotic medication – % of recommended maximum dose above or below the cohort median (75%).

The percentage of the maximum recommended daily dose was calculated for all instances of antipsychotic medication identified through NLP. Maximum recommended daily doses were extracted from the British National Formulary^23^ and the Maudsley prescribing guidelines in Psychiatry.^24^ To ensure data quality and avoid misclassification biases from dosages that were not entered correctly or not identified correctly by NLP, values above 200% of the recommended maximum dose were excluded. For purposes of analysis, we used the highest percentage of maximum recommended daily dose identified for each patient.

### 2.5 Statistical analyses

We compared the demographic, clinical and functional characteristics of patients with and without a history of CSA using chi‐square tests. Next, logistic regression models were fitted for each of the outcome variables (inpatient admission, duration of admission, receipt of antipsychotic medication, antipsychotic dosage). Analyses were adjusted for sex, age at first presentation and ethnicity. Associations were expressed as odds ratios (OR) and their corresponding 95% confidence intervals (CI). Data were analysed using SPSS version25.0.^25^

## 3 RESULTS

We identified 7030 patients with SMI who were in contact with services at C&I NHS FT in the years 2009–2017 and had at least one recorded HoNOS assessment. Fifteen patients were removed from the cohort as they were missing information on the date of first contact with C&I NHS FT, and hence, follow‐up time could not be calculated. An additional 15 were removed as they had a mention of ‘historical sexual abuse’ in their EHR where age at the time of the abuse could not be confirmed, and hence could not be classified as definite childhood events. Hence, the final analytic cohort included 7000 patients with SMI, of which 619 (8.8%) had a recorded history of CSA.

**FIGURE 1:**
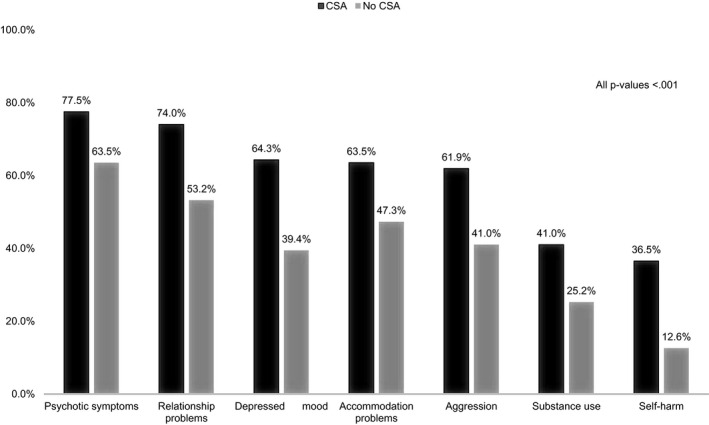
Prevalence of psychopathology and impaired functioning according to recorded history of CSA (from HoNOS)

Patients with a recorded history of CSA were more likely to be female, single, of white ethnic origin than their counterparts without CSA (see Table 1). Comorbid diagnoses of major depressive disorder, post‐traumatic stress disorder and personality disorders (particularly emotionally unstable personality disorder) were all more prevalent in patients with CSA (see Table 2). Higher rates of moderate‐severe positive psychotic symptoms, depressed mood, self‐harm, substance use and aggressive behaviour were also evident in this group, as were problems with relationships and living conditions (see Figure 1).

**TABLE 1:** Demographic characteristic of people with SMI according to recorded history of CSA

	No CSA	CSA	*X* ^2^, *p*‐value
Sex			
M	3609 (56.6%)	224 (36.2%)	94.52, <0.001
F	2772 (43.4%)	395 (63.8%)	
Ethnicity			
White	3462 (54.3%)	411 (66.4%)	64.92, <0.001
Black	1418 (22.2%)	108 (17.4%)	
Asian	485 (7.6%)	25 (4.0%)	
Mixed	257 (4.0%)	43 (6.9%)	
Other	465 (7.3%)	22 (3.6%)	
Unknown	294 (4.6%)	10 (1.6%)	
Marital Status			
Single	4244 (66.5%)	476 (76.9%)	32.27, <0.001
Married	741 (11.6%)	49 (7.9%)	
Divorced	680 (10.7%)	58 (9.4%)	
Widowed	189 (3.0%)	8 (1.3%)	
Unknown	527 (8.3%)	28 (4.5%)	
Social deprivation^a^			
Q1 (least)	957 (15.0%)	83 (13.4%)	9.99, 0.041
Q2	1418 (22.2%)	145 (23.4%)	
Q3	1718 (26.9%)	174 (28.1%)	
Q4 (most)	1729 (27.1%)	183 (29.6%)	
Unknown	559 (8.8%)	34 (5.5%)	

^a^ When removing the ‘unknown’ category, there is no significant difference in the distribution between groups

**TABLE 2:** Comorbid psychiatric diagnoses according to recorded history of CSA

	No CSA	CSA	*X* ^2^, *p*‐value
Major depressive disorder			
No	5895 (92.4%)	536 (86.6%)	25.35, <0.001
Yes	486 (7.6%)	83 (13.4%)	
Post‐traumatic stress disorder			
No	6013 (98.6%)	483 (95.3%)	34.94, <0.001
Yes	92 (1.4%)	29 (4.7%)	
Personality disorders			
No	5976 (94.2%)	498 (78.0%)	221.74, <0.001
Yes	386 (5.8%)	136 (22.0%)	

There was a 2‐fold increase in the odds of inpatient admissions in patients with a history of CSA than in those without (adjusted OR = 1.92, 95% CI: 1.61–2.30), and they were more likely to have spent above 10 days a year as inpatients (adjusted OR = 1.28, 95% CI: 1.04–1.57). Similarly, patients with a history of CSA were more likely to be prescribed antipsychotic medication during the follow‐up period (adjusted OR = 2.48, 95% CI: 1.65–3.66), and more likely to receive over 75% of the maximum recommended daily dosage (adjusted OR = 1.70, 95% CI: 1.42–2.01) as compared to patients with SMI and no recorded history of CSA (see Table 3).

**TABLE 3:** Logistic regression models estimating the effect of CSA on clinical outcomes

	% in No CSA	% in CSA	OR (95% CI)	Adjusted^a^ OR (95% CI)
Inpatient admissions (yes)	51.1%	67.0%	1.95 (1.64–2.32)	1.92 (1.61–2.30)
Above the median inpatient days p/year (10 days)	50.1%	56.9%	1.31 (1.07–1.62)	1.28 (1.04–1.57)
Antipsychotic medication (yes)	89.5%	95.5%	2.48 (1.68–3.65)	2.48 (1.65–3.66)
Above the median maximum recommended daily dose (75%)	47.6%	60.9%	1.72 (1.44–2.04)	1.70 (1.42–2.01)

^a^ Adjusted for sex, age at first presentation and ethnicity.

## 4 DISCUSSION

The current study used routinely recorded data from EHRs to identify a recorded history of CSA in patients with SMI. The study demonstrated that exposure to CSA is associated with more severe symptomatology, impaired functioning, higher rates of comorbidities and negative clinical outcomes in adulthood. This is in line with previous findings, suggesting that CT can have persistent adverse effects on the mental health, social development and well‐being of individuals.^26^

The overall prevalence of CSA in our cohort of patients with SMI was 8.8%. These rates are comparable or slightly lower than self‐reported rates of CSA commonly recorded in community samples^6^ and considerably lower than those reported in a meta‐analysis of patients with psychosis (23.6%, 95% CI: 21.2% to 32.2%).^5^ This is most likely related to the method of identifying CSA used in this study. Unlike the meta‐analysis, and most previous studies, we used string‐matching text searches in EHRs rather than psychometric instruments to detect a record of CSA. It has been reported that the rates of childhood trauma documented in mental health notes may be a significant under‐representation of the actual rates.^27^, ^28^ Reasons for this include (a) clinicians not enquiring about CT, (b) clinicians not documenting reports of CT and (c) a lack of disclosure by individuals attending the mental health services of CT.^27^

Similar to previous studies in the general population^2^ and among people with SMI,^8^, ^15^ the prevalence of CSA was significantly higher among women than men. This difference may be because of either higher occurrence of CSA among women, or to the reluctance of men to disclose CSA. Similarly, the higher prevalence of CSA among patients from white ethnic origin may represent a real difference in rates or may be an artefact of reporting biases (eg, more underreporting of minorities to service providers^29^) or racial biases in detection or recording of CSA. Comorbid diagnoses of major depressive disorder, post‐traumatic stress disorder and personality disorders were all more prevalent in patients with a history of CSA. Similarly, a moderate‐severe rating on all clinical and functional items extracted from the HoNOS was more common among patients with CSA than among those without documented CSA. These include positive psychotic symptoms, depressed mood, self‐harm, substance use, aggressive behaviour, problems with relationships and problems with living conditions. This is in line with the conclusions of a review by Grubaugh et al.^7^ finding that, among individuals with SMI, childhood trauma is correlated with alcohol and/or drug use, transient living conditions, the additional presence of a personality disorder, suicidality and self‐injurious behaviours, measures of hostility and anger, and indices of social, occupational and community functioning.

One of the most consistent findings in the literature is that patients with a history of CSA report more severe delusions and hallucinations. Several theories have been proposed to explain this association, including that post‐traumatic stress disorder and psychosis involve shared mechanisms; that hallucinations are variations of post‐traumatic intrusions; and that delusions may develop as a result of childhood trauma via biased threat beliefs, stemming from negative beliefs about self and others.^30^

The odds of having an inpatient admission were almost twice as high in patients with a history of CSA, and they were 30% more likely to have been hospitalised more than 10 days per year than patients without CSA. Previous studies have also found that patients with SMI and a history of CT, particularly childhood sexual and physical abuse, have a greater number of psychiatric hospitalisations.^31^, ^32^ Additionally, while the vast majority of patients in this study were receiving antipsychotic medication, those with a history of CSA were 2.5 times more likely to receive such medication and the prescribed doses were higher. While the evidence on the use of psychotropic medication in people with SMI who have experienced CSA is scarce, Schneeberger et al.^33^ reported findings similar to ours whereby patients with SMI who had experienced childhood trauma received higher doses of antipsychotic medication. The higher rates of positive psychotic symptoms discussed earlier may be associated with a more severe and treatment refractory form of illness, hence explaining the higher dosage of antipsychotics prescribed in this population.

Taken together, the findings of this study suggest that while identification of CSA instances through EHRs most likely leads to an under‐representation of cases, CSA is clearly associated with more severe psychopathology, poorer functioning and worse clinical outcomes in adulthood. Future studies should build on these initial findings and examine specific comorbid diagnoses and symptom profiles, which are associated with a childhood history of CSA in patients with SMI.

### 4.1 Strengths and limitations

This study uses a novel approach to identify CSA instances in free‐text fields of EHRs. This approach affords the opportunity to examine the course of illness and longer‐term outcomes among patients with SMI in a naturalistic mental healthcare setting. Our data provided access to a very large cohort of patients with SMI. Hence, our findings are directly relevant to real‐world clinical settings providing care for patients with SMI in the UK (except prison and secure hospital settings where mental illness, substance misuse and childhood trauma may all serve as predisposing factors to offence‐related behaviour).

Our study has several limitations. First, the data were obtained from routine electronic clinical records and were not collected for research purposes. Second, we used a simple string‐matching technique to identify cases of CSA in free‐text fields of EHRs. While the PPV of our search algorithm was high, this does not account for the possibility that some cases may have been missed (as suggested by the lower prevalence of CSA as compared to previous studies). It is possible that there are additional search terms that were not used in this study, which may have led to the identification of additional cases. The under‐reporting and/or under‐recording of CSA in EHRs may have led to an underestimation of the true association between CSA and the outcomes described in this study. Third, patients with more or longer admissions might have had more opportunities to have CSA recorded in their EHRs, partially explaining this association. However, this cannot be true for the increased antipsychotic prescribing, dose and HoNOS items. Finally, our search strategy did not allow us to extract information on the age or duration of sexual abuse, variables which have been shown to be associated with the clinical presentation of psychotic disorders.

To conclude clinicians working with patients with SMI (namely, non‐organic psychotic disorders and bipolar disorder) should be trained and skilled at assessing childhood adversity as this may have prognostic and treatment implications. While the observational nature of this study does not allow us to make treatment recommendations, clinicians and researchers should consider evidence‐based trauma‐focused interventions, such as eye movement desensitisation and reprocessing (EMDR) and cognitive analytic therapy (CAT), when forming treatment plans for people with a history of CSA. These interventions have been proven to be effective in reducing trauma symptoms,^34^ psychological distress,^35^ dissociation^36^ and negative emotions^37^ in both the short and long term.

This study demonstrates the potential of using novel approaches for identification of CSA in routinely collected electronic clinical records. The secondary use of data collected in EHRs can dramatically increase the breadth and depth of information available for research.^38^ While structured fields lend themselves well to computational analysis, free‐text fields represent an estimated 60–70% of the data in EHRs.^19^ Thus, harnessing different techniques for data mining de‐identified free text can provide a broader and richer picture of what is documented in EHRs. Applying data‐driven approaches, such as machine learning and natural language processing, to large data sets is a promising avenue for cost‐effective and reliable research on childhood trauma.

### PEER REVIEW

The peer review history for this article is available at https://publons.com/publon/10.1111/acps.13302.

## Data Availability

The data used in this work have been obtained from the Clinical Record Interactive Search (CRIS), a system which has been implemented at the Camden & Islington NHS Foundation Trust (C&I). It provides authorised researchers with regulated access to anonymised information extracted from patient electronic health records. CRIS is governed by a strict information governance scheme, which forbids anyone except for authorised researchers from accessing its records. Access to CRIS is restricted to (1) C&I employees or (2) those having an honorary contract or letter of access from the Trust. Once an honorary contract is established, researchers can only access CRIS once they submit a research project proposal through the CRIS Project Application form. The form is available here: http://www.candi.nhs.uk/health‐professionals/research/ci‐research‐database/researchers‐and‐clinicians For further details, contact: researchdatabase@candi.nhs.uk.

## APPENDIX 1 Terms used for the identification of childhood sexual abuse in clinical notes

childhood sexual abuse

childhood history of sexual abuse

childhood Hx of sexual abuse

csa

sexual* abuse* at age [0–9]

history of sexual abuse at age [0–9]

hx of sexual abuse at age [0–9]

sexual abuse in childhood

sexual* abuse* as a child

sexual* abuse* between age* [0–9] and [0–9]

childhood experience* of sexual abuse

sexually abused from age [0–9]

1. * wildcard allowing for any characters after the defined string
2. [0–9] allowing for any numerical character
3. Some of the search terms were also used with variations including ‘the’ and ‘of’ (eg ‘history of sexual abuse at the age of [0–9]’)
